# Spinal Epidural Fat as an Imaging Biomarker of Visceral Obesity: An MRI-Based Quantitative Analysis

**DOI:** 10.3390/diagnostics15192490

**Published:** 2025-09-29

**Authors:** Nicola Marrone, Gabriele Bilancia, Domenico Romeo, Valerio D’Agostino, Federico Ponti, Francesca Salamanna, Amandine Crombé, Paolo Spinnato

**Affiliations:** 1 Diagnostic and Interventional Radiology, IRCCS Istituto Ortopedico Rizzoli, 40136 Bologna, Italy; nicomarrone95@gmail.com (N.M.);; 2 Department of Medical and Surgical Sciences (DIMEC), University of Bologna, 40138 Bologna, Italy; 3 Surgical Sciences and Technologies, IRCCS Istituto Ortopedico Rizzoli, 40136 Bologna, Italy; 4 Department of Diagnostic Imaging, Gustave Roussy Institute, 94800 Villejuif, France

**Keywords:** magnetic resonance imaging, epidural space, spinal epidural lipomatosis, metabolic syndrome, abdominal obesity metabolic syndrome, visceral adipose tissue

## Abstract

**Background/Objectives**: Spinal epidural lipomatosis (SEL) is increasingly recognized as a possible radiological indicator of Metabolic Syndrome (MS) and visceral adiposity. However, the precise relationship between visceral adiposity and the accumulation of epidural fat (EF) remains unclear. This study aimed to investigate the association between visceral adipose tissue (VAT) and EF thickness using quantitative MRI analyses. **Methods**: We retrospectively reviewed all MRI scans performed at our institution over a 7-month period, from May to November 2024. Two radiologists measured and recorded the VAT maximum antero-posterior diameter at the L3 level, EF maximum diameter at the L5-S1 level, spinal canal antero-posterior diameter at the L5-S1 level, and subcutaneous fat (SF) when included in the MRI images (at the L3 level) in all the MRI scans. **Results**: A cohort of 516 patients was collected (320 women and 196 men; mean age 57.31 ± 18.45 years old). In 508 patients (98.4%) SF and VAT were both measurable, while in 8 patients VAT only was assessable on MRI scans. Pearson correlation identified significant associations between EF and VAT thickness (correlation coefficient > 20%; *p* < 0.05). A linear regression model confirmed a significant, albeit modest, positive relationship between VAT and EF (R^2^ = 5.4%). A multivariate regression model incorporating age, sex, spinal canal size, VAT, and SF improved the explanatory power (adjusted R^2^ = 16.7%), with VAT, spinal canal diameter, and age emerging as significant predictors of EF (*p* < 0.001). **Conclusions**: Our study revealed in a large cohort of patients that EF and VAT are directly associated. On the other hand, SF resulted in not being associated with EF. These findings support the emerging concept that SEL can be a radiological phenotype of visceral obesity and, by extension, of MS. Integrating EF measurement into standard MRI interpretation may facilitate the early detection of SEL and offer additional insights into patients’ underlying metabolic profile.

## 1. Introduction

Spinal epidural lipomatosis (SEL) is a pathological condition characterized by the excessive deposition of unencapsulated adipose tissue in the epidural space, most commonly affecting the lumbosacral region [1]. When fat accumulation becomes significant, SEL may lead to compression of adjacent neural structures, resulting in a wide spectrum of neurological symptoms including low back pain, neurogenic claudication, radiculopathy, and—in severe cases—cauda equina syndrome [2,3].

**Figure 1:**
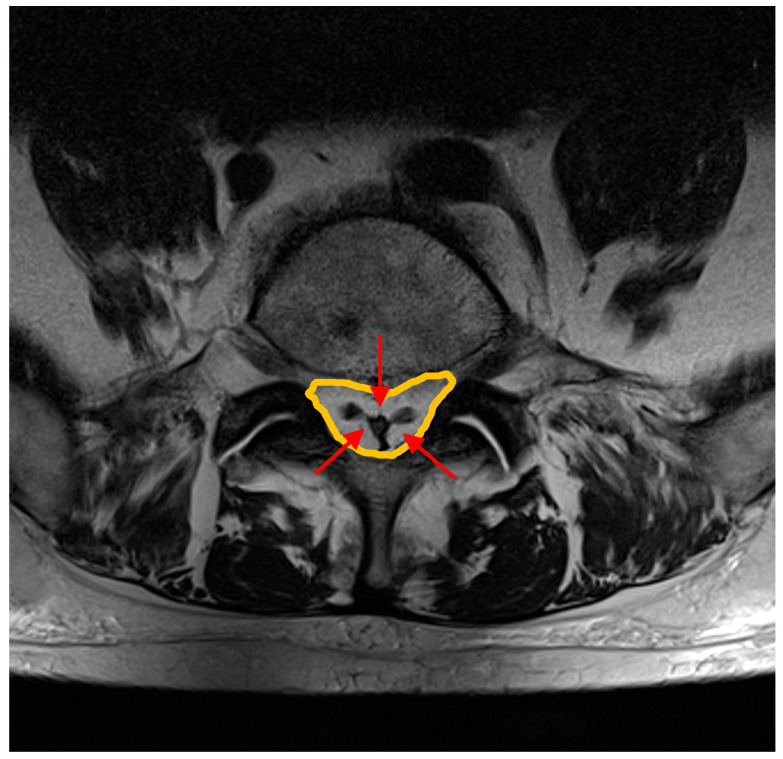
MRI axial T1-weighted sequence shows the presence of excessive accumulation of epidural fat within the spinal epidural space (orange line), resulting in compression of the dural sac and thus leading to a “Y” shaped configuration (indicated by the red arrows); a well-known MRI sign of severe spinal epidural lipomatosis which has implication on systemic health assessment due to the possible linkage with metabolic syndrome. In this case, the patient comes to our attention for low back pain and neurogenic claudication of the lower limbs for about a year, with difficulty climbing the stairs.

Traditionally considered as a rare clinical entity, SEL is now increasingly recognized [4,5,6,7,8,9,10,11,12,13,14,15,16,17,18,19,20,21,22,23,24,25], primarily due to the widespread use of Magnetic Resonance Imaging (MRI), which is considered the gold standard for non-invasive diagnosis [26,27,28,29,30] (Figure 1).

Despite its clinical implications, SEL remains markedly underreported, with previous studies indicating a diagnostic rate as low as 8% in routine lumbar MRI reports [31]. Recent epidemiological studies have reported a higher prevalence than previously estimated, reaching up to 16.7% in specific patient cohorts undergoing lumbar spine MRI [3]. Although SEL can occasionally be diagnosed on Computed Tomography (CT) due to the negative attenuation values (attenuation < 0 Hounsfield Unit) of adipose tissue, MRI remains the most sensitive modality for detecting EF and for assessing the severity and distribution of SEL [31]. As the recognition of SEL has increased, so too has our understanding of its underlying risk factors.

Although long-term corticosteroid exposure, whether endogenous [32,33,34,35,36,37] or exogenous [38,39,40,41,42,43,44,45,46,47,48,49,50,51], has historically been considered as the primary etiologic factor, growing evidence now implicates obesity and, more broadly, metabolic syndrome as the leading contributors to SEL development [3,28,31,52,53,54,55,56,57,58,59].

Metabolic Syndrome (MS) is diagnosed according to the International Diabetes Federation (IDF 2005) criteria, which require central visceral obesity (waist circumference ≥ 94 cm for men or ≥80 cm for women, or BMI > 30 kg/m^2^) plus at least two of the following: hypertension (≥130/85 mmHg or on antihypertensive treatment), hypertriglyceridemia (≥150 mg/dL or on treatment), low HDL cholesterol (<40 mg/dL in men, <50 mg/dL in women or on treatment), and elevated fasting glucose (≥100 mg/dL or treatment) [60,61,62,63,64].

Given the central role of visceral adiposity in the definition of MS, particular attention was paid to Visceral Adipose Tissue (VAT), which is known to contribute to a metabolic state of chronic low-grade inflammation and its complications, including insulin resistance, type 2 diabetes, cardiovascular disease, liver disease, cancer, and neurodegeneration [3,62,65].

Additionally, recent studies have demonstrated a significant correlation between markers of VAT and SEL, suggesting that SEL may represent an unrecognized clinical manifestation of MS in the spine [3,58,59,66]. In detail, Ishihara et al. found that SEL was significantly associated with high body mass index (BMI), abdominal circumference, and VAT deposition, reporting an odds ratio of 3.9 in patients diagnosed with MS compared to controls [67].

In light of this emerging evidence, this study aims to systematically investigate the relationship between epidural fat (EF) and VAT accumulation. A secondary aim of the study was to assess the possible correlation between EF and subcutaneous fat (SF).

Early recognition of SEL on MRI examinations could offer a valuable opportunity to detect subclinical metabolic disorders, with potential critical implications for cardiovascular risk stratification and the implementation of targeted preventive strategies.

## 2. Materials and Methods

We retrospectively reviewed all the lumbosacral MRIs performed in our single Institution during a 7-month period (May–November 2024). The database included patients who underwent lumbosacral MRI performed on the same 1.5T scanner (Signa, GE Healthcare^®^, Chicago, IL, USA) for various clinical indications. This retrospective observational study was conducted in line with Helsinki’s declaration criteria and was approved by the local IRB.

All the patients with lumbosacral MRI examination, including MRI sequences adequate for L5-S1 level assessment (T1w and T2w sagittal and axial-oblique plane), and for VAT assessment from MR-localizer sequences. MRIs with artifacts (including metal implants) or MR-localizer sequences unable to include the whole VAT diameter at the L3 level were excluded.

Few clinical variables were recorded: age and sex.

**Figure 2:**
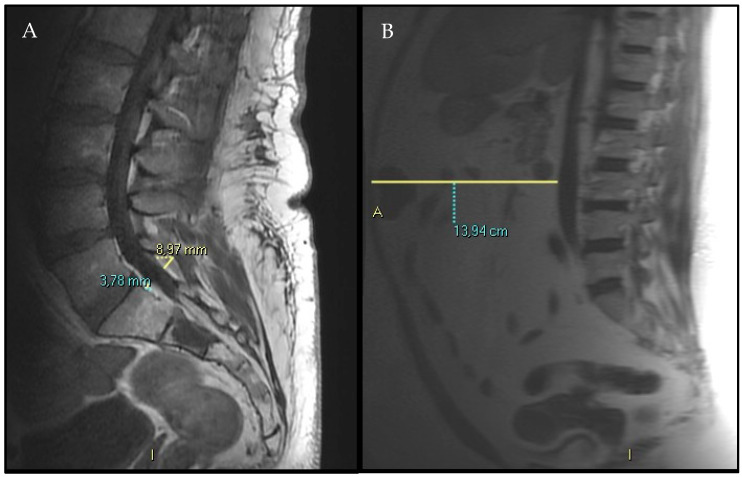
Exemplification of epidural fat (EF) and visceral adipose tissue (VAT) measurement on MRI studies (**A**). T1-weighted sagittal sequences were used to assess anterior and posterior EF thickness within the SC, measured in a plane parallel and tangent to the superior endplate of the S1 vertebral body. (**B**) MR-localized sequences were employed to evaluate VAT deposition, which is the distance between the abdominal muscular fascia and the anterior wall of the abdominal aorta at the L3 vertebral level. Please note that commas (,) have been used as decimal signs in the figures measurements.

Several imaging variables were analyzed and collected on lumbosacral MRIs:

- EF (anterior plus posterior) diameter at S1 superior endplate level following Borrè’s reproducible method [2] (Figure 2, panel A).
- Spinal canal antero-posterior diameter (SC), at the same level.
- VAT anterior–posterior max diameter at L3 level represented by the distance between the abdominal muscular fascia and the anterior wall of the abdominal aorta at the L3 vertebral level; MR-localizer sequences were used to achieve this aim (Figure 2, panel B).
- SF anterior–posterior diameter has been measured and recorded as well, whether it was comprised in the MR-localizer sequences at the same level (L3), anterior subcutaneous fat pad.

All the imaging analyses were performed by two radiologists (N.M. and D.R.) with 3 years of experience in the field of musculoskeletal and general radiology. We followed the EF measurements criteria set by Borrè et al. in their previous research, in which inter- and intra-observer variability had already been performed with substantial to excellent agreement in a large cohort of 2528 patients [2].

The above-mentioned clinical and radiological data have been correlated to check their association with VAT and EF (and SAT when available). Moreover, patients have been subdivided into three categories of VAT accumulation: (i) low (VAT diameter <5 cm), (ii) intermediate (VAT diameter 5–7 cm), (iii) high (VAT diameter >7 cm). The differences in EF values in the three VAT categories have been investigated.

### Statistical Analysis

Statistical analyses were conducted using RStudio (R version 4.2.0). Descriptive statistics were first applied, including histograms and boxplots. The normality of the distributions was assessed using the Shapiro–Wilk test and Q-Q plots. Inferential statistics included a one-way ANOVA to assess differences in EF thickness among VAT-defined subgroups, followed by Tukey’s HSD post hoc test to identify pairwise significance. Pearson’s correlation coefficients were calculated to evaluate linear associations between variables. A univariate linear regression model was employed to quantify the relationship between the selected independent variables and the dependent variable EF. To improve explanatory power, a multivariate linear regression model was constructed.

*p*-values < 0.05 were considered statistically significant. Moreover, the significant *p*-values have been subdivided into three classes as follows: (i) *p* < 0.001 (highly significant), (ii) *p* < 0.01 (moderately significant), (iii) *p* < 0.05 (mildly significant)

## 3. Results

### 3.1. Patients’ Population and Selection

Among 535 MRIs analyzed, 19 (3.5%) have been excluded because the key elements of the study (EF, VAT, SC) were not correctly assessable for different reasons (e.g., metal artifacts, movement artifacts, inadequate MRI field of view).

The final dataset included 516 MRIs of 516 patients (320 women and 196 men; mean Age 57.31 ± 18.45 years). In 508 of those patients (508/516—98.4%), the MRI field of view of MR-localizer sequences permits the correct measurement of both SF and VAT. In the remaining 8 patients (1.6%), VAT was only assessable on MRI scans.

### 3.2. Visceral and Epidural Fat Associations

The distribution of VAT and EF was analyzed across the study cohort to identify potential patterns. Descriptive statistics of the VAT values distribution through the cohort of patients, including minimum, maximum, mean, and median values, along with interquartile ranges (all expressed in millimeters), were reported to better characterize data variability (Table 1).

**Figure 3:**
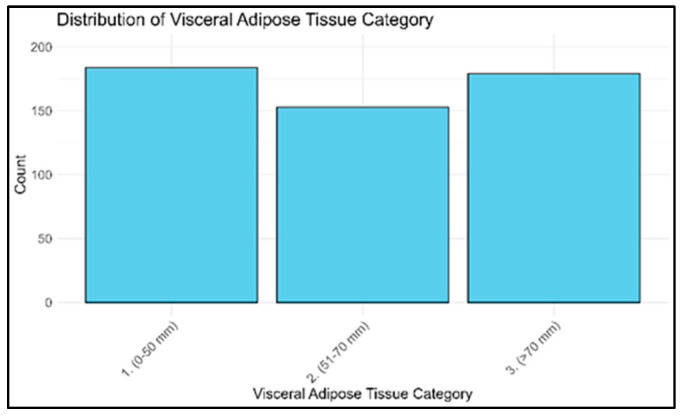
Histogram of patients’ distribution among the defined visceral adipose tissue categories (1) low, (2) intermediate, and (3) high.

The cohort was divided into three VAT subgroups: (i) low, (ii) intermediate, and (iii) high VAT accumulation—(see ‘materials and methods’ Section 2). In Figure 3, the stratification of patients according to different amounts of VAT is reported.

**Figure 4:**
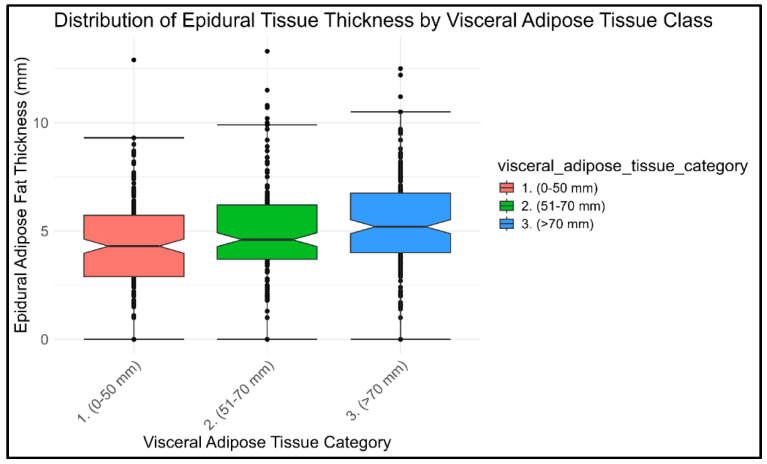
Box Plot of the distribution of the EF in the different visceral adipose tissue (VAT) Categories (1. Low, 2. Intermediate, and 3. High VAT amount).

A box plot was constructed to illustrate the distribution of EF across the VAT classes: (1) low, (2) intermediate, (3) high. The plot reveals an increasing mean EF thickness with higher VAT classes, suggesting a potential positive association between the two variables (Figure 4).

A one-way ANOVA demonstrated a statistically significant difference in EF thickness among the three VAT subgroups (F = 9.57; *p* = 0.00005). Post hoc analysis using Tukey’s HSD test revealed that EF thickness was significantly greater in patients with moderate and high VAT measurements (VAT subgroups i and ii—between 51 and 70 mm—and >70 mm, compared to those with VAT 0–50 mm (*p* = 0.18), as shown in Table 2.

These results show a measurable variation in EF thickness across VAT-defined subgroups, with the most pronounced differences occurring between the lowest VAT category and the two higher ranges. The stratification by VAT thickness enabled a clear comparison of EF values across the population.

#### 3.2.1. Correlation Analysis

**Figure 5:**
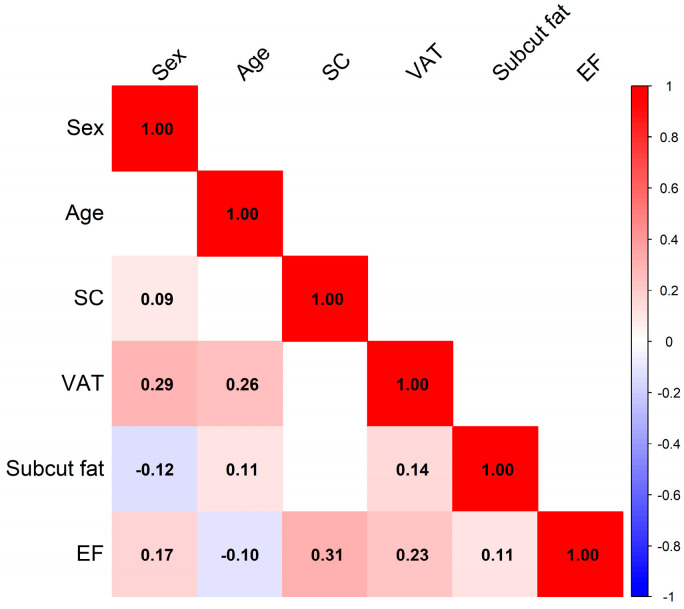
This figure illustrates the correlations between sex, age, spinal canal diameter (SC), visceral adipose tissue (VAT), subcutaneous fat, and epidural fat (EF). Colors indicate the strength and direction of the associations: red for positive correlations, blue for negative ones. The more intense the color, the stronger the correlation. The numbers within each cell represent the Pearson correlation coefficient, which ranges from −1 (perfect inverse association) to +1 (perfect direct association). Only statistically significant correlations (*p* < 0.05) are displayed. Key findings: Spinal canal diameter (SC) shows a weak positive correlation only with sex (r = 0.09). Visceral adipose tissue (VAT) is positively correlated with both sex (r = 0.29) and age (r = 0.26), suggesting that men and older individuals tend to have higher visceral fat. - Subcutaneous fat is negatively correlated with sex (r = −0.12), and positively with age (r = 0.11) and VAT (r = 0.14), indicating that subcutaneous fat increases slightly with age and is modestly associated with visceral fat. Epidural fat (EF) shows multiple associations: it is positively correlated with sex (r = 0.17), SC diameter (r = 0.31), VAT (r = 0.23), and subcutaneous fat (r = 0.11), while showing a weak negative correlation with age (r = −0.10). These findings suggest that epidural fat may be influenced by both anatomical and adipose-related factors.

A correlation matrix was computed using Pearson’s correlation coefficient to assess the relationships among key variables per subject. Only statistically significant correlations (*p* < 0.05) were included in the visual output (Figure 5).

The strongest correlation with EF thickness was observed for SC diameter and VAT. Both variables showed correlation coefficients exceeding 20%, indicating a potentially meaningful association with EF accumulation.

#### 3.2.2. Linear Regression Model

**Figure 6:**
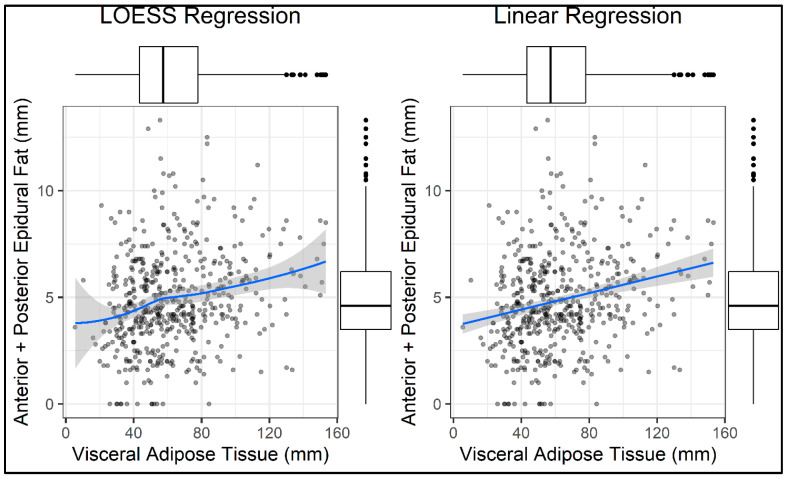
This figure displays two scatter plots illustrating the relationship between visceral adipose tissue (*X*-axis, in mm) and the combined anterior and posterior epidural fat (*Y*-axis, in mm): Left panel: LOESS regression (Locally Estimated Scatterplot Smoothing), a non-parametric method used to explore potential non-linear patterns in the data. The blue curve represents the locally fitted model, with the shaded region indicating the 95% confidence interval. Right panel: Linear regression, showing a straight-line fit to the same data, with its corresponding confidence interval. Each black dot represents an individual data point. Boxplots along both axes summarize the distribution of each variable, aiding in the visualization of spread, central tendency, and potential outliers. LOESS regression is typically applied when the underlying relationship between variables may not be strictly linear. In this case, however, the LOESS curve closely resembles the linear regression line, with no substantial deviations or curvature. This visual similarity suggests that the association between visceral adipose tissue and epidural fat can be adequately modeled using a simple linear approach, without the need for more complex, non-linear modeling.

The relationship between VAT (independent variable) and the combined anterior + posterior EF thickness (dependent variable) was assessed through an Ordinary Least Squares (OLS) linear regression model (Figure 6).

Prior to fitting the model, a Loess smoother plot was used to evaluate the linearity assumption, which was visually supported by the earlier box plot. No substantial deviation from linearity was found, confirming the appropriateness of the linear model. Therefore, the coefficients have been estimated by using the OLS method.

A simple linear regression analysis was performed to assess the relationship between VAT and EF thickness, with results summarized in Table 3.

The model revealed a statistically significant positive association between VAT and EF, with an estimated regression coefficient of 0.02 mm (standard error = 0.004; *p* = 7.75 × 10^−7^), indicating that for each additional millimeter of VAT, EF thickness increases by approximately 0.02 mm. Despite the statistical significance of both coefficients, the model’s explanatory power was modest. The adjusted R^2^ value was 0.054, meaning that VAT alone accounted for approximately 5.4% of the variability in EF thickness. The model’s overall significance was confirmed by the F-statistic of 29.31 and its associated *p*-value of 9.48 × 10^−8^, supporting the conclusion that the observed association is unlikely to be due to chance.

While the strength of the association is relatively weak, the direction and statistical significance of the findings support the hypothesis that visceral adiposity contributes to EF accumulation. These results suggest a potential link between systemic metabolic status and local fat deposition within the SC.

### 3.3. Multivariate Regression Model

Given that VAT alone explained only a limited proportion of the variability in EF thickness, a multivariate linear regression model was employed to assess the combined contribution of additional predictors. Independent variables were selected based on their statistically significant correlations with the dependent variable and included: Age, sex, SC diameter, VAT, and SF. In addition, an interaction term between SC diameter and VAT (canal × VAT) was introduced to evaluate whether the association between VAT and EF thickness varied according to canal size.

The analysis identified Age, SC diameter, and VAT thickness as significant predictors of EF thickness (all *p* < 0.001). Notably, Age demonstrated a negative association (β = −0.02), suggesting a decrease in EF thickness with advancing age. In contrast, SC diameter (β = 0.17) and VAT thickness (β = 0.02) were positively associated with EF thickness.

Other variables, including Sex and SF, did not reach statistical significance in the multivariate analysis (*p* = 0.15 and *p* = 0.16, respectively), suggesting a marginal or negligible effect in this model. The interaction term (canal × VAT) was also non-significant (*p* = 0.23), indicating that the effect of VAT on EF thickness does not appear to be modulated by SC size.

Multicollinearity diagnostics indicated no significant concerns, with tolerance values above 0.85 for all variables, supporting the stability and independence of predictors within the model. The model explained 17.75% of the variance in EF thickness (R^2^ = 0.1775), with an adjusted R^2^ of 0.167, indicating moderate explanatory capacity and good generalizability. The model’s overall significance was supported by an F-statistic of 16.84 and a *p*-value < 2.2 × 10^−16^, confirming strong global significance.

These results indicate that SC diameter, visceral adiposity, and Age are independent and significant predictors of EF thickness. While SF showed only marginal contributions, the non-significant interaction term suggests that VAT and SC diameter exert independent effects on EF. Although the explained variance remains modest, the model provides valuable insight into the multifactorial determinants of SEL and suggests potential avenues for improved risk stratification through combined clinical and imaging parameters.

Table 4 presents the results of the univariate analysis for the selected independent variables, while Table 5 summarizes the findings from the multivariate regression model.

## 4. Discussion

The present study explored the association between visceral adiposity and epidural adipose tissue accumulation using both univariate and multivariate statistical approaches, with the aim of elucidating whether increased VAT—an established marker of MS, could serve as a predictor of SEL. Our findings demonstrate a significant and quantifiable relationship between VAT and EF deposition, further supporting the concept of SEL as a radiological phenotype of visceral obesity and, by extension, of MS.

Unlike prior investigations by Morishita et al. [26] and Ishihara et al. [67], which primarily established the existence of an association between visceral adiposity and EF accumulation, our study provides a more detailed and stratified analysis in a larger series (Vs 218 and 324 patients, respectively).

A combination of distribution analysis, inferential statistics, and both univariate and multivariate regression models has been used to characterize this association in a radiologically stratified cohort. Descriptive stratification of the population based on VAT thickness allowed for an unbiased comparative framework.

The box plot analysis illustrated a clear gradient: patients with greater VAT thickness exhibited proportionally increased EF thickness. This trend was statistically confirmed through one-way ANOVA, which revealed significant differences in EF thickness across the three VAT subgroups (F = 9.57; *p* = 0.00005). Post hoc comparisons using Tukey’s HSD test indicated that EF thickness was significantly greater in patients with VAT measurements of 51–70 mm (*p* = 0.04) and >70 mm (*p* < 0.001) compared to those with VAT ≤ 50 mm. These findings suggest a dose-dependent relationship between visceral adiposity and EF proliferation, reinforcing prior literature on the association between obesity and SEL [3,28,31,52,55].

Correlation analysis using Pearson’s coefficient identified VAT and spinal canal (SC) diameter as the two strongest correlates of EF thickness, both demonstrating correlation coefficients > 0.20. This suggests that while VAT is a key systemic driver of EF deposition, anatomical parameters such as SC size may modulate the extent to which EF can accumulate. This finding is consistent with existing literature indicating that SEL more frequently manifests in patients with borderline or congenitally narrowed canals, thereby increasing the risk of symptomatic neural compression [58,59].

Linear regression model confirmed the presence of a statistically significant association between VAT and EF thickness (*p* < 0.001), although the explained variance was modest (adjusted R^2^ = 5.2%). This suggests that VAT, while relevant, is only one of several determinants of EF burden.

To better understand EF variability, the multivariate regression model—incorporating Age, SC diameter, VAT, and Subcutaneous Adipose Tissue (SAT)—offered improved explanatory power (adjusted R^2^ = 16.7%). Within this model, VAT (*p* < 0.001), Age (*p* < 0.001), and SC diameter (*p* < 0.001) emerged as independent predictors of EF thickness, while SAT was not statistically significant.

The strongest contribution of SC diameter compared to other variables suggests that local anatomical constraints could magnify the effects of systemic adiposity, potentially exacerbating EF-related compression. The lack of significance for the interaction term (canal × VAT) indicates that the relationship between VAT and EF is likely additive rather than multiplicative and is not significantly altered by SC size in this sample. Similarly, the absence of a significant contribution from SAT supports the notion that visceral rather than peripheral adiposity is the key systemic driver of EF proliferation.

An intriguing finding of our analysis was the negative association between EF thickness and age, in contrast with the general expectation that adiposity increases over the years. To the best of our knowledge, no studies on this have been specifically carried out previously. One hypothesis could be adipose tissue distribution shifts over time: visceral fat tends to increase, while EF may decline due to changes in SC anatomy or reduced marrow adipogenesis. Ishihara et al. demonstrated that EF correlates more with visceral adiposity and MS rather than with age itself, which showed no correlation in their study. In addition to biological mechanisms, referral and selection biases may play a role. Elderly patients undergoing lumbar MRI are frequently imaged for degenerative spinal stenosis or postsurgical conditions, which do not necessarily overlap with obesity-related EF accumulation. Conversely, younger obese patients might undergo imaging only when symptomatic, leading to a relative overrepresentation of high EF volumes in younger age categories.

Beyond statistical associations, our findings hold several practical implications for clinical practice. Firstly, the routine quantification of EF on lumbar MRI could serve as a non-invasive screening tool for identifying patients at increased risk of MS. For instance, a patient referred for MRI due to back pain who demonstrates disproportionate EF deposition may warrant further evaluation for hypertension, dyslipidemia, or impaired glucose tolerance, even in the absence of overt symptoms.

Furthermore, EF measurement may help guide patient management and follow-up protocols. Patients with marked EF accumulation, especially in combination with exceeding VAT deposition, could benefit from early referral to endocrinology or metabolic clinics for lifestyle modification, weight reduction, or pharmacologic interventions targeting insulin resistance. In such contexts, EF may represent not only a marker of systemic adiposity but also a potential surrogate endpoint to monitor therapeutic response.

Finally, EF quantification could be incorporated into risk stratification strategies for spinal canal stenosis. In patients with borderline canal dimensions, the presence of excessive EF may accelerate the onset of symptomatic compression. Identifying such individuals early could influence surgical timing or prompt preventive measures aimed at mitigating disease progression.

In the future, serial MRIs could track changes in EF following weight loss or other treatments, aiming to assess if the decrease in VAT that can be achieved with treatments, or a change in lifestyle (e.g., diet, physical exercise) would even reflect in a parallel reduction in EF amount. In the authors’ personal experience (P.S. and F.P.) we noticed a unidirectional simultaneous change in EF and VAT amount for patients with lumbosacral MRIs follow-up controls over time.

**Figure 7:**
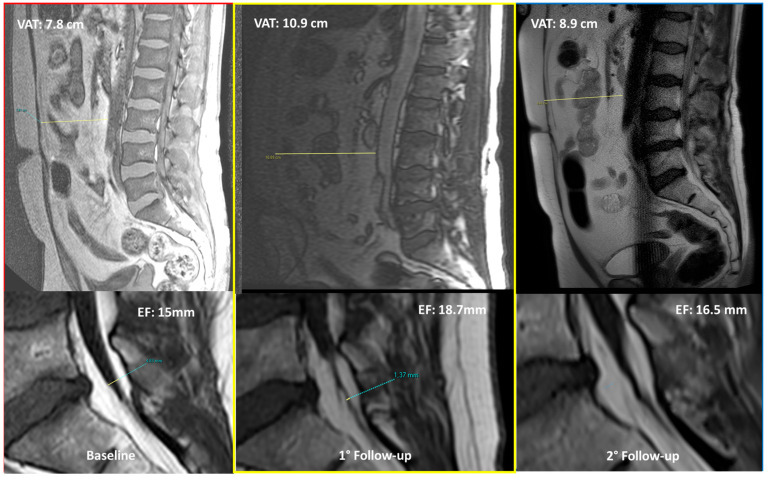
MRI controls over time (baseline, 1° follow-up, and 2° follow-up) in a middle-aged man affected by spinal epidural lipomatosis (with neurologic symptoms) and metabolic syndrome (with moderate overweight)—MR-localizer sequences (up), T1w sagittal sequences (down). VAT = visceral adipose tissue (cm). EF = epidural fat (mm). Patients’ weight at the moment of follow-up controls (1° MRI = 76 kg, 2° MRI = 87 kg, 3° MRI = 83 kg).

**Figure 8:**
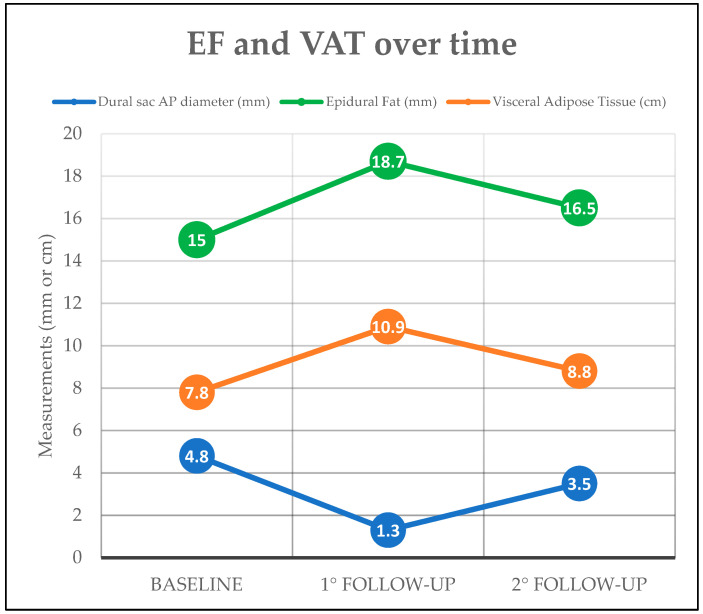
Measured values of visceral adipose tissue (VAT—cm), and epidural fat (EF—mm) over time in 3 subsequent MRI controls. The graph shows a clear association between changes in EF and VAT over time. Dural Sac Antero-posterior (AP) Diameter changes are also reported and inversely correlated with EF amount.

An exemplifying case concerning EF and VAT changes over time is provided in Figure 7 and Figure 8.

Although our analyses demonstrated a statistically significant association between VAT and EF, the explained variance was relatively low. In the univariate model, VAT accounted for only ~5% of the variability in EF, while the multivariate model increased the explained variance to ~16%. These findings indicate that EF accumulation is influenced by multiple factors beyond VAT, and that VAT alone has limited predictive value for EF thickness.

According to this, several limitations of this study should be acknowledged. Key anthropometric parameters such as body mass index (BMI), waist circumference, and the patient’s weight were not available. These measures are strongly associated with visceral adiposity and MS, and their absence limits our ability to contextualize EF accumulation within broader body composition patterns. Secondly, we lacked data on metabolic biomarkers (e.g., fasting glucose, HbA1c, lipid profile, insulin levels, and inflammatory markers), which could have strengthened the pathophysiological link between EF, VAT deposition, and systemic metabolic status. Moreover, a detailed history of corticosteroid exposure—both systemic and local (epidural injections)—was not available for the cohort. Given that chronic corticosteroid therapy is a well-established risk factor for SEL, its absence may be considered a major confounding factor.

Together, these limitations constrain the generalizability of our findings and suggest that future studies should adopt a multidisciplinary design, integrating radiologic quantification of EF with anthropometric, biochemical, and pharmacological data. Such an approach would more robustly delineate the independent contribution of visceral adiposity and MS to EF deposition.

Another methodological limitation lies in the fact that VAT, EF, and SF were quantified using maximum diameters rather than volumetric analysis. This approach was chosen because it is simple, fast, and easily reproducible in routine radiological practice, without requiring advanced post-processing software or extended reporting time. Nevertheless, this method may be less robust than volumetric quantification, as it does not capture the three-dimensional distribution and heterogeneity of adipose tissue. Future studies should incorporate fully volumetric MRI-based fat quantification to validate and potentially refine the associations observed here.

During the preparation of this work, the authors used Generative Artificial Intelligence (GAI) to improve language editing, only for the Introduction section. After using this tool, the authors reviewed and edited the content as needed and took full responsibility for the content of the publication, according to the GAMER statement [68].

## 5. Conclusions

This study demonstrates a statistically significant association between VAT and EF accumulation in the lumbar spine, as assessed by MRI. Although the correlation is modest in strength, the findings reinforce the hypothesis that SEL is not merely a localized spinal condition but a radiological expression of systemic visceral adiposity.

Multivariate analysis confirmed that VAT, SC diameter, and Age are independent predictors of EF thickness, while SAT and sex play only a marginal role. These results underscore the need for increased radiological attention to EF measurements, particularly in patients with central obesity or metabolic risk factors.

Given the diagnostic underreporting of SEL in routine MRI practice, this study highlights the value of incorporating EF quantification into standard spinal assessments. Such radiologic vigilance may not only aid in identifying a potential cause of SC stenosis but also serve as a non-invasive marker of underlying metabolic dysfunction.

Future studies incorporating volumetric quantification and integrating imaging biomarkers with clinical and metabolic data are warranted to further define the diagnostic and prognostic role of SEL within the broader context of systemic disease.

## Figures and Tables

**Table 1:** Descriptive statistics of the VAT values distribution through the cohort of patients.

Min.	1st Qu.	Median	Mean	3rd Qu.	Max.
5.50	43.10	56.80	62.10	77.12	153.20

**Table 2:** Comparison of EF Thickness Across VAT Subgroups.

VAT Group Comparison	Mean Difference in EF Thickness (mm)	95% Confidence Interval	*p*-Value
0–50 mm vs. 51–70 mm	0.58	0.01–1.16	0.04 *
0–50 mm vs. >70 mm	1.02	0.47–1.58	0.00005 ***
51–70 mm vs. >70 mm	0.44	−0.14–1.02	0.18

*** *p* < 0.001 (highly significant); ** *p* < 0.01 (moderately significant); * *p* < 0.05 (mildly significant).

**Table 3:** Univariate regression model results between visceral adipose tissue (VAT, independent variable) and epidural fat (EF, dependent variable).

Variable	Estimate (mm)	Std. Error	*p*-Value
Intercept	3.65	0.25	<2 × 10^−16^ ***
VAT	0.02	0.004	7.75 × 10^−7^ ***

*** *p* < 0.001 (highly significant); ** *p* < 0.01 (moderately significant); * *p* < 0.05 (mildly significant).

**Table 4:** Univariate regression model results. Each row reports the outcome of a separate linear regression performed for each selected independent variable. Statistically significant results are highlighted in bold.

Variable	Estimate	Std. Error	*p*-Value	Adjusted R^2^
**Age**	**−0.013**	**0.006**	0.006 ***	**0.008**
**Sex**	**0.81**	**0.20**	8.02 × 10^−5^ ***	**0.03**
Subcutaneous Adipose Tissue (mm)	0.026	0.01	0.01 *	0.011
**Spinal Canal Diameter (mm)**	**0.175**	**0.024**	0.02 *	**0.09**

*** *p* < 0.001 (highly significant); ** *p* < 0.01 (moderately significant); * *p* < 0.05 (mildly significant).

**Table 5:** Multivariate regression model results illustrating the linear relationship between selected independent variables and the dependent variable EF. Statistically significant results are highlighted in bold.

Variable	Estimate	Std. Error	*p*-Value	Tolerance
**(Intercept)**	**1.68**	**0.56**	0.003 **	**–**
**Age**	**−0.02**	**0.01**	2.94 × 10^−5^ ***	**0.95**
Sex	0.30	0.21	0.15	0.87
**Spinal Canal Diameter (mm)**	**0.17**	**0.02**	9.59 × 10^−12^ ***	**0.96**
**Visceral Adipose Tissue (mm)**	**0.02**	**0.004**	7.75 × 10^−6^ ***	**0.87**
Subcutaneous Adipose Tissue (mm)	0.01	0.01	0.16	0.94
canal × VAT interaction	−0.12	0.10	0.23	0.97

*** *p* < 0.001 (highly significant); ** *p* < 0.01 (moderately significant); * *p* < 0.05 (mildly significant).

## Data Availability

Further information and additional data can be requested by contacting the corresponding author (Paolo Spinnato, paolo.spinnato1982@gmail.com or paolo.spinnato@ior.it).
